# *trans*-Di­chlorido­bis­(dimethyl sulfoxide-κ*O*)bis­(4-fluoro­benzyl-κ*C* ^1^)tin(IV): crystal structure and Hirshfeld surface analysis

**DOI:** 10.1107/S2056989017005072

**Published:** 2017-04-07

**Authors:** Nur Adibah Binti Mohd Amin, Rusnah Syahila Duali Hussen, See Mun Lee, Nathan R. Halcovitch, Mukesh M. Jotani, Edward R. T. Tiekink

**Affiliations:** a Department of Chemistry, University of Malaya, 50603 Kuala Lumpur, Malaysia; b Research Centre for Crystalline Materials, School of Science and Technology, Sunway University, 47500 Bandar Sunway, Selangor Darul Ehsan, Malaysia; c Department of Chemistry, Lancaster University, Lancaster LA1 4YB, United Kingdom; d Department of Physics, Bhavan’s Sheth R. A. College of Science, Ahmedabad, Gujarat 380001, India

**Keywords:** crystal structure, organotin, C—H⋯F inter­actions, Hirshfeld surface analysis

## Abstract

The Sn^IV^ atom in the title diorganotin compound, [Sn(C_7_H_6_F)_2_Cl_2_(C_2_H_6_OS)_2_], is located on a centre of inversion, resulting in the C_2_Cl_2_O_2_ donor set having an all-*trans* disposition of like atoms. The coordination geometry approximates an octa­hedron. The crystal features C—H⋯F, C—H⋯Cl and C—H⋯π inter­actions, giving rise to a three-dimensional network. The respective influences of the Cl⋯H/H⋯Cl and F⋯H/H⋯F contacts to the mol­ecular packing are clearly evident from the analysis of the Hirshfeld surface.

## Abstract 2

The octa­hedrally coordinated Sn^IV^ atom in [Sn(C_7_H_6_F)_2_Cl_2_(C_2_H_6_OS)_2_] is located on a centre of inversion so the resulting donor C_2_Cl_2_O_2_ donor set is all-*trans*. The three-dimensional mol­ecular packing is sustained by C—H⋯F, C—H⋯Cl and C—H⋯π inter­actions.

## Chemical context   

The structural chemistry of organotin(IV) compounds with multidentate Schiff base ligands has been of inter­est since the observation of the diversity in their supra­molecular association patterns (Teoh *et al.*, 1997 ▸; Dey *et al.*, 1999 ▸). Typically, these multidentate ligands bind to the tin atom through the phenolic-O, imine-N, oxime-O or even oxime-N atoms. In view of this, the coordination of these multidentate ligands to (organo)tin may lead to more thermodynamically stable organotin complexes, in contrast to those with monodentate ligands (Vallet *et al.*, 2003 ▸; Contreras *et al.*, 2009 ▸), a feature which could potentially be useful in catalytic studies (Yearwood *et al.*, 2002 ▸). In consideration of this and as part of on-going work with multidentate ligands of organotin compounds (Lee *et al.*, 2004 ▸), an attempt to synthesize an adduct of the potentially tetra­dentate Schiff base *N,N*-1,1,2,2-di­nitrile­vinyl­ene*bis*(5-bromo­salicylaldiminato) with di(*p*-fluoro­benz­yl)tin(IV) dichloride was made.
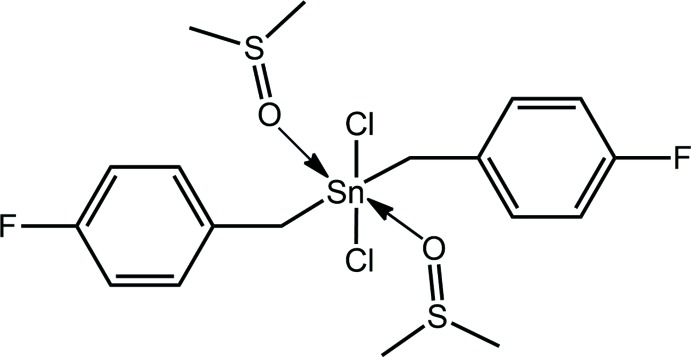


The complex was obtained as an orange powder and was successfully characterized using various spectroscopic methods including ^1^H NMR spectroscopy. Upon inter­action with DMSO-*d*_6_, in the context of NMR studies, colourless crystals were obtained after several weeks standing. The formation of the new title compound, (I), is likely due to degradation of the complex while stored in the NMR tube. In the present contribution, the crystal and mol­ecular structures of (I) are described as well as a detailed analysis of the inter­molecular association through a Hirshfeld surface analysis.

## Structural commentary   

**Figure 1:**
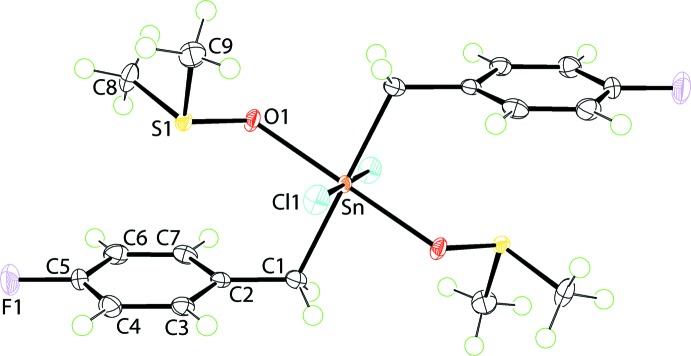
The mol­ecular structure of (I), showing the atom-labelling scheme and displacement ellipsoids at the 70% probability level. The Sn^IV^ atom lies on a centre of inversion; unlabelled atoms are related by the symmetry operation 1 − *x*, 1 − *y*, 1 − *z*.

The mol­ecular structure of (I), Fig. 1 ▸, has the Sn^IV^ atom situated on a crystallographic centre of inversion. The Sn^IV^ atom is coordinated by monodentate ligands, *i.e*. chloride, sulfoxide-O and methyl­ene-C atoms. From symmetry, each donor is *trans* to a like atom resulting in an all-*trans*-C_2_Cl_2_O_2_ donor set about the Sn^IV^ atom. The donor set defines a distorted octa­hedral geometry owing, in part, to the disparate Sn—donor atom bond lengths, Table 1 ▸. The angles about the Sn^IV^ atom differ relatively little from the ideal octa­hedral angles with the maximum deviation of *ca* 6° noted for the C1—Sn—O1 angle, Table 1 ▸.

## Supra­molecular features   

**Figure 2:**
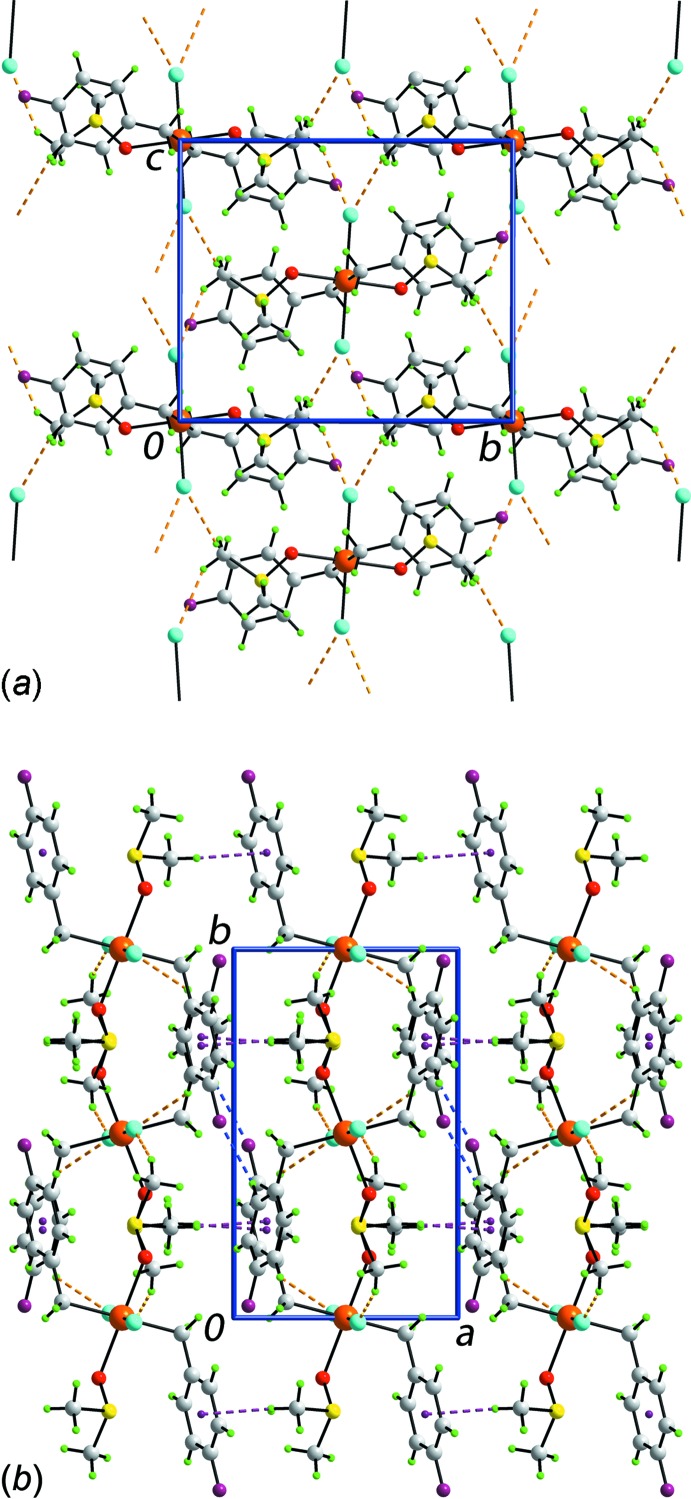
The mol­ecular packing in (I): (*a*) supra­molecular layer in the *bc* plane sustained by C—H⋯Cl inter­actions and (*b*) a view of the unit-cell contents in projection down the *c* axis. The C—H⋯Cl, C—H⋯F and C—H⋯π inter­actions are shown as orange, blue and purple dashed lines, respectively.

The mol­ecular packing in (I) comprises C—H⋯F, C—H⋯Cl and C—H⋯π inter­actions which combine to generate a three-dimensional network, Table 2 ▸. The chloride atom participates in phenyl-C6—H⋯Cl1 and methyl-C8—H⋯Cl1 inter­actions. As each chloride atom is involved in two C—H⋯Cl inter­actions and there are two chloride atoms per mol­ecule, the C—H⋯Cl inter­actions extend laterally to give rise to a supra­molecular layer in the *bc* plane, Fig. 2 ▸ *a*. Layers are connected along the *a* axis by phenyl-C3—H⋯F1 and methyl-C9—H⋯π(phen­yl) inter­actions to consolidate the mol­ecular packing, Fig. 2 ▸ *b*.

## Hirshfeld surface analysis   

**Figure 3:**
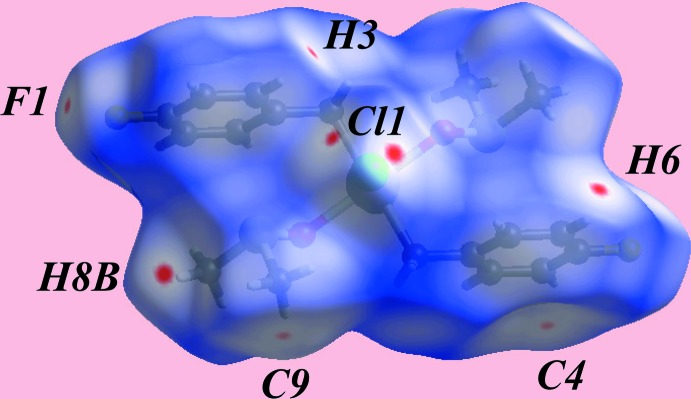
A view of the Hirshfeld surface for (I) mapped over *d*_norm_ over the range −0.049 to 1.356 au.

**Figure 4:**
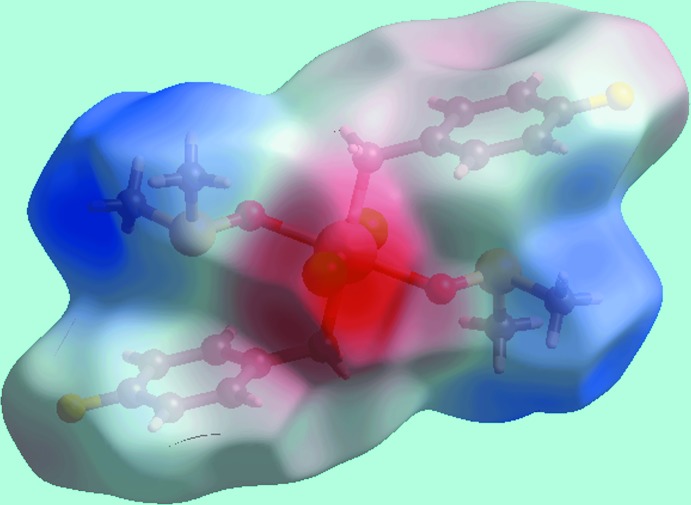
A view of the Hirshfeld surface for (I) mapped over the electrostatic potential in the range ±0.095 au.

**Figure 5:**
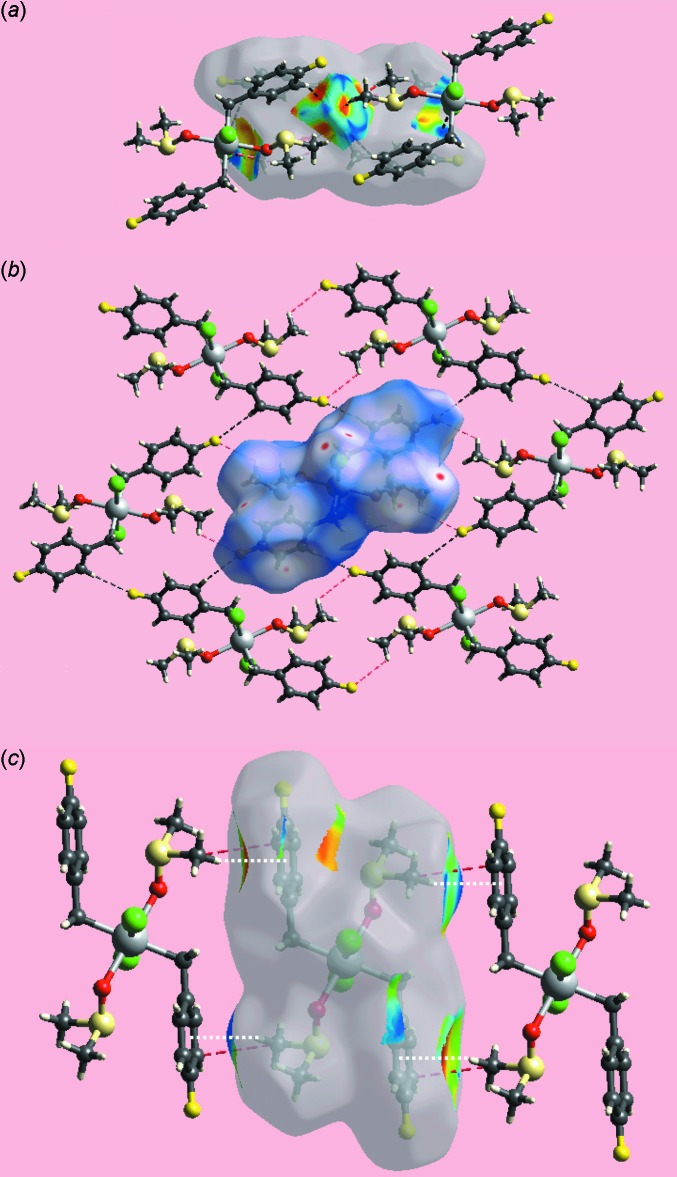
Views of the Hirshfeld surfaces about a reference mol­ecule mapped over (*a*) shape-index, (*b*) *d*_norm_ and (*c*) shape-index, highlighting (*a*) C—H⋯F and short inter­atomic F⋯H/H⋯F contacts as black and red dashed lines, respectively, (*b*) C—H⋯Cl and short inter­atomic Cl⋯H/H⋯Cl contacts as black and red dashed lines, respectively, and (*c*) C—H⋯π and short inter­atomic C⋯C contacts as white and red dashed lines, respectively.

The Hirshfeld surface analysis on the structure of (I) provides more insight into the mol­ecular packing and was performed as described recently (Wardell *et al.*, 2016 ▸). It is evident from the bright-red spots appearing near the chloride and fluoride atoms on the Hirshfeld surface mapped over *d*_norm_ in Fig. 3 ▸ that these atoms play a significant role in the mol­ecular packing. Thus, the bright-red spots near phenyl-H6, methyl-H8*B* and a pair near Cl1 in Fig. 3 ▸ indicate the presence of bifurcated C—H⋯Cl inter­actions formed by each of the chloride atoms. Similarly, the pair of red spots near phenyl-H3 and F1 atoms are associated with the donor and acceptor of C—H⋯F inter­actions, respectively. The donors and acceptors of C—H⋯Cl and C—H⋯F inter­actions are also represented with blue (positive potential) and red regions (negative potential), respectively, on the Hirshfeld surface mapped over the electrostatic potential in Fig. 4 ▸. In addition to above, the Cl1 and F1 atoms also participate in short inter­atomic contacts with methyl-H atoms, Table 3 ▸. The presence of faint-red spots near the phenyl-C4 and methyl-C9 atoms in Fig. 3 ▸ indicate their participation in a short inter­atomic C⋯C contact, Table 3 ▸, which compliments the methyl-C—H⋯π(phen­yl) contact described above. The presence of the C—H⋯π inter­action is also evident from the view of Hirshfeld surface mapped over the electrostatic potential around participating atoms, Fig. 4 ▸; the donors and acceptors of these inter­actions are viewed as the convex surface around atoms of the methyl-C9 groups and the concave surface above the (C2–C7) phenyl ring, respectively. The immediate environments about a reference mol­ecule within *d*_norm_- and shape-index-mapped Hirshfeld surfaces highlighting the various C—H⋯Cl, C—H⋯F and C—H⋯π inter­actions are illustrated in Fig. 5 ▸ *a*–*c*, respectively.

**Figure 6:**
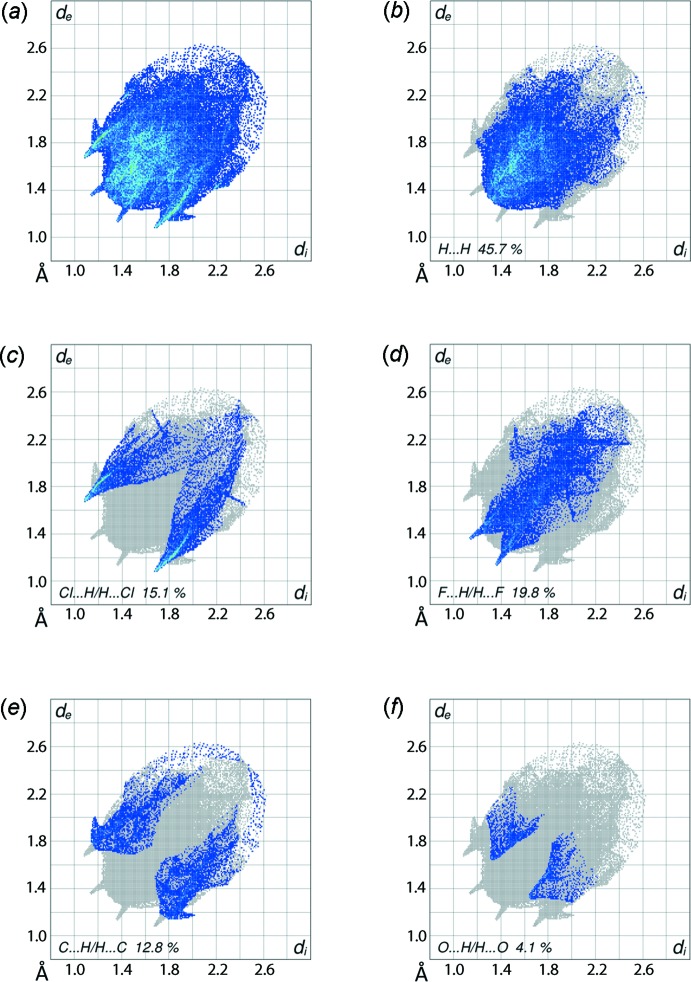
Fingerprint plots for (I): (*a*) overall and those delineated into (*b*) H⋯H, (*c*) Cl⋯H/H⋯Cl, (*d*) F⋯H/H⋯F, (*e*) C⋯H/H⋯C and (*f*) O⋯H/H⋯O contacts.

The overall two-dimensional fingerprint plot and those delineated into H⋯H, Cl⋯H/H⋯Cl, F⋯H/H⋯F, C⋯H/H⋯C and O⋯H/H⋯O contacts (McKinnon *et al.*, 2007 ▸) are illustrated in Fig. 6 ▸ *a*–*f*, respectively, and their relative contributions to the Hirshfeld surfaces are summarized in Table 4 ▸. It is clear from the fingerprint plot delineated into H⋯H contacts, Fig. 6 ▸ *b*, that although these contacts have the greatest contribution, *i.e*. 45.7%, to the Hirshfeld surface, the dispersion forces acting between them keep these atoms at the distances greater than the sum of their van der Waals radii, hence they do not contribute significantly to the mol­ecular packing. The comparatively greater contribution of F⋯H/H⋯F contacts to the Hirshfeld surface *cf*. Cl⋯H/H⋯Cl contacts, Table 4 ▸, is due to the relative positions of the chloride and fluoride atoms in the mol­ecule, the fluoride atoms being at the extremities and the chloride atoms near the tin(IV) atom. However, the Cl⋯H/H⋯Cl contacts have a greater influence on the mol­ecular packing as viewed from the delineated fingerprint plot in Fig. 6 ▸ *c*. The forceps-like distribution of points in the plot with tips at *d*_e_ + *d*_i_ ∼2.8 Å result from the bifurcated C—H⋯Cl inter­actions, and points at positions less than the sum of their van der Waals radii are ascribed to the short inter­atomic Cl⋯H/H⋯Cl contacts, the green appearance due to high density of inter­actions. Similarly, a pair of short spikes at *d*_e_ + *d*_i_ ∼2.5 Å in the fingerprint plot delineated into F⋯H/H⋯F contacts, Fig. 6 ▸ *d*, are indicative of inter­molecular C—H⋯F inter­actions with the short inter­atomic F⋯H/H⋯F contacts merged within the fingerprint plot. It is important to note from the fingerprint plot delineated into C⋯H/H⋯C contacts, Fig. 6 ▸ *e*, that even though their inter­atomic distances are equal to or greater than the sum of their van der Waals radii, *i.e*. 2.9 Å, the 12.8% contribution from these to the Hirshfeld surfaces are indicative of the presence of C—H⋯π inter­actions in the structure. This is also justified from the presence of short inter­atomic C⋯C contacts, Fig. 5 ▸ *c* and Table 3 ▸. The 4.1% contribution from O⋯H/H⋯O contributions to Hirshfeld surfaces, Fig. 6 ▸ *f*, and the small contributions from the other contacts listed in Table 2 ▸ have a negligible effect on the packing.

## Database survey   

There are three related structures of the general formula *R*_2_Sn*X*_2_(DMSO)_2_ in the crystallographic literature (Groom *et al.*, 2016 ▸). Key bond angles for these are listed in Table 5 ▸. The Me_2_SnBr_2_(DMSO)_2_ compound (Aslanov *et al.*, 1978 ▸) is analogous to (I) in that the Sn^IV^ atom is located on a centre of inversion and hence, is an all-*trans* isomer. The two remaining structures have a different arrangements of donor atoms with the common feature being the *trans*-disposition of the Sn-bound organic groups, with the halides and DMSO-O atoms being mutually *cis*, i.e. *R* = Me and *X* = Cl (Aslanov *et al.*, 1978 ▸; Isaacs & Kennard, 1970 ▸) and *R* = Ph and *X* = Cl (Sadiq-ur-Rehman *et al.*, 2007 ▸). Clearly, further studies are required to ascertain the factor(s) determining the adoption of one coordination geometry over another.

## Synthesis and crystallization   

All chemicals and solvents were used as purchased without purification. Di(*p*-fluoro­benz­yl)tin dichloride was prepared in accordance with the literature method (Sisido *et al.*, 1961 ▸). All reactions were carried out under ambient conditions. The melting point was determined using an Electrothermal digital melting point apparatus and was uncorrected. The IR spectrum was obtained on a Perkin Elmer Spectrum 400 FT Mid-IR/Far-IR spectrophotometer in the range 4000 to 400 cm^−1^. The ^1^H NMR spectrum was recorded at room temperature in CDCl_3_ solution on a Jeol ECA 400 MHz FT–NMR spectrometer.

*N*,*N*′-1,1,2,2-Di­nitrile­vinyl­enebis(5-bromo­salicylaldiminato) (1.0 mmol, 0.401 g; prepared by the condensation reaction between di­amino­maleo­nitrile and 5-bromo­sal­icyl­alde­hyde in a 2:1 molar ratio in ethanol) and tri­ethyl­amine (1.0 mmol, 0.14 ml) in ethyl acetate (25 ml) was added to di(*p*-fluoro­benz­yl)tin dichloride (1.0 mmol, 0.183 g) in ethyl acetate (10 ml). The resulting mixture was stirred and refluxed for 4 h. The filtrate was evaporated until a dark-orange precipitate was obtained. The precipitate was dissolved in DMSO-*d*_6_ solution in a NMR tube for ^1^H NMR spectroscopic characterization. After the analysis, the tube was set aside for a month and colourless crystals of (I) suitable for X-ray crystallographic studies were obtained from the slow evaporation. Yield: 0.060 g, 11%; m.p: 399 K. IR (cm^−1^): 1595(m) ν(C=C), 1504(s) ν(S=O), 1161(*m*), 578(*w*), 508(*m*) ν(Sn—O). ^1^H NMR (in CDCl_3_): 6.90–7.11, 7.35–7.40 (*m*, 8H, aromatic-H), 3.11 (*s*, 6H, –CH_3_), 2.17 (*m*, 4H, –CH_2_).

## Refinement details   

Crystal data, data collection and structure refinement details are summarized in Table 6 ▸. Carbon-bound H-atoms were placed in calculated positions (C—H = 0.95–0.99 Å) and were included in the refinement in the riding model approximation, with *U*_iso_(H) set to 1.2–1.5*U*_eq_(C).

## Supplementary Material

Crystal structure: contains datablock(s) I, global. DOI: 10.1107/S2056989017005072/wm5381sup1.cif

Structure factors: contains datablock(s) I. DOI: 10.1107/S2056989017005072/wm5381Isup2.hkl

CCDC reference: 1541712

Additional supporting information:  crystallographic information; 3D view; checkCIF report

## Figures and Tables

**Table 1:** Selected geometric parameters (Å, °)

Sn—C1	2.1628 (16)	Sn—Cl1	2.5599 (4)
Sn—O1	2.2332 (11)		
			
C1—Sn—O1	95.99 (5)	C1—Sn—Cl1^i^	89.95 (5)
C1—Sn—Cl1	90.05 (5)	O1—Sn—Cl1	90.44 (3)
C1—Sn—O1^i^	84.01 (5)	O1—Sn—Cl1^i^	89.56 (3)

Symmetry code: (i) 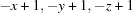.

**Table 2:** Hydrogen-bond geometry (Å, °) *Cg*1 is the centroid of the C2–C7 ring.

*D*—H⋯*A*	*D*—H	H⋯*A*	*D*⋯*A*	*D*—H⋯*A*
C3—H3⋯F1^ii^	0.95	2.49	3.333 (2)	147
C6—H6⋯Cl1^iii^	0.95	2.77	3.6129 (18)	148
C8—H8*B*⋯Cl1^iv^	0.98	2.76	3.6379 (17)	150
C9—H9*C*⋯*Cg*1^v^	0.98	2.67	3.3887 (19)	130

Symmetry codes: (ii) 
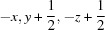
; (iii) 
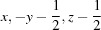
; (iv) 
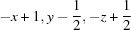
; (v) 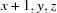.

**Table 3:** Summary of short inter­atomic contacts (Å) in (I)

Contact	distance	symmetry operation
C4⋯C9	3.371 (2)	−1 + *x*, *y*, *z*
F1⋯H8*A*	2.66	1 − *x*, −*y*, 1 − *z*
Cl1⋯H9*B*	2.91	1 − *x*, 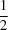 + *y*, 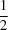 − *z*

Note: (*a*) the Sn^IV^ atom is located on a centre of inversion.

**Table 4:** Percentage contribution of inter­atomic contacts to the Hirshfeld surface for (I)

Contact	percentage contribution
H⋯H	45.7
Cl⋯H/H⋯Cl	15.1
F⋯H/H⋯F	19.8
C⋯H/H⋯C	12.8
O⋯H/H⋯O	4.1
S⋯H/H⋯S	1.7
Cl⋯F/F⋯Cl	0.6
C⋯Cl/Cl⋯C	0.1
F⋯F	0.1

**Table 5:** Selected geometric parameters (Å, °) for mol­ecules of the general formula *R*_2_Sn*X*_2_(DMSO)_2_

Compound	*X*—Sn—*X*	O—Sn—O	C—Sn—C	Reference
Me_2_SnBr_2_(DMSO)_2_	180	180	180	Aslanov *et al.* (1978 ▸)
Me_2_SnCl_2_(DMSO)_2_	95.2 (3)	83.7 (5)	172.7 (3)	Aslanov *et al.* (1978 ▸)
Ph_2_SnCl_2_(DMSO)_2_	97.43 (3)	79.34 (9)	172.17 (14)	Sadiq-ur-Rehman *et al.* (2007 ▸)
(4-FC_6_H_4_CH_2_)_2_SnCl_2_(DMSO)_2_	180	180	180	This work

**Table 6:** Experimental details

Crystal data	
Chemical formula	[Sn(C_7_H_6_F)_2_Cl_2_(C_2_H_6_OS)_2_]
*M*_r_	564.08
Crystal system, space group	Monoclinic, *P*2_1_/*c*
Temperature (K)	100
*a*, *b*, *c* (Å)	8.2363 (1), 12.7020 (2), 11.4038 (1)
β (°)	110.391 (2)
*V* (Å^3^)	1118.28 (3)
*Z*	2
Radiation type	Cu *K*α
μ (mm^−1^)	13.28
Crystal size (mm)	0.24 × 0.12 × 0.10

Data collection	
Diffractometer	Agilent SuperNova, Dual, Cu at zero, AtlasS2
Absorption correction	Multi-scan (*CrysAlis PRO*; Rigaku Oxford Diffraction, 2015 ▸)
*T*_min_, *T*_max_	0.636, 1.000
No. of measured, independent and observed [*I* > 2σ(*I*)] reflections	7792, 2292, 2228
*R*_int_	0.020
(sin θ/λ)_max_ (Å^−1^)	0.631

Refinement	
*R*[*F* ^2^ > 2σ(*F* ^2^)], *wR*(*F* ^2^), *S*	0.018, 0.046, 1.08
No. of reflections	2292
No. of parameters	126
H-atom treatment	H-atom parameters constrained
Δρ_max_, Δρ_min_ (e Å^−3^)	0.35, −0.76

Computer programs: *CrysAlis PRO* (Rigaku Oxford Diffraction, 2015 ▸), *SHELXS* (Sheldrick, 2008 ▸), *SHELXL2014* (Sheldrick, 2015 ▸), *ORTEP-3 for Windows* (Farrugia, 2012 ▸), *DIAMOND* (Brandenburg, 2006 ▸) and *publCIF* (Westrip, 2010 ▸).

## supplementary crystallographic information

### Crystal data

**Table:**

[Sn(C_7_H_6_F)_2_Cl_2_(C_2_H_6_OS)_2_]	*F*(000) = 564
*M**_r_* = 564.08	*D*_x_ = 1.675 Mg m^−^^3^
Monoclinic, *P*2_1_/*c*	Cu *K*α radiation, λ = 1.54184 Å
*a* = 8.2363 (1) Å	Cell parameters from 6127 reflections
*b* = 12.7020 (2) Å	θ = 5.4–76.3°
*c* = 11.4038 (1) Å	µ = 13.28 mm^−^^1^
β = 110.391 (2)°	*T* = 100 K
*V* = 1118.28 (3) Å^3^	Prism, colourless
*Z* = 2	0.24 × 0.12 × 0.10 mm

### Data collection

**Table:**

Agilent SuperNova, Dual, Cu at zero, AtlasS2 diffractometer	2292 independent reflections
Radiation source: micro-focus sealed X-ray tube, SuperNova (Cu) X-ray Source	2228 reflections with *I* > 2σ(*I*)
Mirror monochromator	*R*_int_ = 0.020
ω scans	θ_max_ = 76.5°, θ_min_ = 5.4°
Absorption correction: multi-scan (CrysAlis PRO; Rigaku Oxford Diffraction, 2015)	*h* = −10→9
*T*_min_ = 0.636, *T*_max_ = 1.000	*k* = −15→15
7792 measured reflections	*l* = −14→14

### Refinement

**Table:**

Refinement on *F*^2^	0 restraints
Least-squares matrix: full	Hydrogen site location: inferred from neighbouring sites
*R*[*F*^2^ > 2σ(*F*^2^)] = 0.018	H-atom parameters constrained
*wR*(*F*^2^) = 0.046	*w* = 1/[σ^2^(*F*_o_^2^) + (0.0233*P*)^2^ + 0.7527*P*] where *P* = (*F*_o_^2^ + 2*F*_c_^2^)/3
*S* = 1.08	(Δ/σ)_max_ < 0.001
2292 reflections	Δρ_max_ = 0.35 e Å^−^^3^
126 parameters	Δρ_min_ = −0.76 e Å^−^^3^

### Special details

**Table:**

Geometry. All esds (except the esd in the dihedral angle between two l.s. planes) are estimated using the full covariance matrix. The cell esds are taken into account individually in the estimation of esds in distances, angles and torsion angles; correlations between esds in cell parameters are only used when they are defined by crystal symmetry. An approximate (isotropic) treatment of cell esds is used for estimating esds involving l.s. planes.

### Fractional atomic coordinates and isotropic or equivalent isotropic displacement parameters (Å^2^)

**Table:**

	*x*	*y*	*z*	*U*_iso_*/*U*_eq_	
Sn	0.5000	0.5000	0.5000	0.00673 (6)	
Cl1	0.44665 (6)	0.48762 (3)	0.26545 (4)	0.01508 (10)	
S1	0.55778 (5)	0.24628 (3)	0.43015 (3)	0.00881 (9)	
F1	0.06456 (15)	0.03219 (9)	0.34347 (11)	0.0248 (2)	
O1	0.60349 (15)	0.33583 (9)	0.52752 (10)	0.0106 (2)	
C1	0.2332 (2)	0.45714 (13)	0.46551 (16)	0.0130 (3)	
H1A	0.2054	0.4746	0.5410	0.016*	
H1B	0.1583	0.5013	0.3962	0.016*	
C2	0.1878 (2)	0.34437 (13)	0.43339 (15)	0.0098 (3)	
C3	0.1093 (2)	0.31260 (13)	0.30871 (15)	0.0111 (3)	
H3	0.0838	0.3637	0.2440	0.013*	
C4	0.0679 (2)	0.20772 (14)	0.27783 (15)	0.0134 (3)	
H4	0.0147	0.1868	0.1930	0.016*	
C5	0.1058 (2)	0.13487 (13)	0.37280 (16)	0.0140 (3)	
C6	0.1822 (2)	0.16192 (14)	0.49747 (16)	0.0144 (3)	
H6	0.2062	0.1101	0.5613	0.017*	
C7	0.2229 (2)	0.26727 (14)	0.52668 (15)	0.0121 (3)	
H7	0.2758	0.2873	0.6118	0.014*	
C8	0.6371 (2)	0.13286 (13)	0.52466 (15)	0.0154 (3)	
H8A	0.7576	0.1447	0.5786	0.023*	
H8B	0.6312	0.0718	0.4708	0.023*	
H8C	0.5662	0.1197	0.5766	0.023*	
C9	0.7181 (2)	0.25482 (14)	0.35875 (16)	0.0159 (3)	
H9A	0.7035	0.3209	0.3118	0.024*	
H9B	0.7055	0.1952	0.3017	0.024*	
H9C	0.8335	0.2530	0.4235	0.024*	

### Atomic displacement parameters (Å^2^)

**Table:**

	*U*^11^	*U*^22^	*U*^33^	*U*^12^	*U*^13^	*U*^23^
Sn	0.00785 (9)	0.00543 (9)	0.00565 (8)	0.00005 (4)	0.00078 (6)	−0.00080 (4)
Cl1	0.0208 (2)	0.0153 (2)	0.00719 (18)	0.00143 (14)	0.00249 (16)	0.00013 (13)
S1	0.00860 (18)	0.00802 (18)	0.00877 (16)	0.00079 (13)	0.00171 (14)	−0.00157 (13)
F1	0.0250 (6)	0.0089 (5)	0.0328 (6)	−0.0026 (4)	0.0006 (5)	−0.0014 (5)
O1	0.0135 (6)	0.0067 (5)	0.0095 (5)	0.0016 (4)	0.0012 (4)	−0.0021 (4)
C1	0.0082 (7)	0.0131 (9)	0.0172 (8)	−0.0007 (6)	0.0039 (6)	−0.0030 (6)
C2	0.0059 (7)	0.0112 (8)	0.0122 (7)	0.0000 (6)	0.0032 (6)	−0.0012 (6)
C3	0.0087 (7)	0.0129 (8)	0.0105 (7)	0.0005 (6)	0.0018 (6)	0.0023 (6)
C4	0.0115 (8)	0.0152 (9)	0.0117 (7)	−0.0004 (6)	0.0020 (6)	−0.0028 (6)
C5	0.0110 (8)	0.0078 (8)	0.0211 (8)	−0.0014 (6)	0.0030 (6)	−0.0018 (6)
C6	0.0117 (8)	0.0151 (8)	0.0152 (8)	0.0015 (6)	0.0032 (6)	0.0065 (6)
C7	0.0089 (8)	0.0176 (8)	0.0090 (7)	−0.0006 (6)	0.0022 (6)	0.0000 (6)
C8	0.0228 (9)	0.0080 (8)	0.0141 (8)	0.0027 (6)	0.0049 (7)	0.0002 (6)
C9	0.0166 (9)	0.0175 (9)	0.0167 (8)	0.0005 (6)	0.0097 (7)	−0.0019 (6)

### Geometric parameters (Å, º)

**Table:**

Sn—C1	2.1628 (16)	C3—C4	1.390 (2)
Sn—C1^i^	2.1628 (16)	C3—H3	0.9500
Sn—O1	2.2332 (11)	C4—C5	1.375 (2)
Sn—O1^i^	2.2332 (11)	C4—H4	0.9500
Sn—Cl1^i^	2.5599 (4)	C5—C6	1.383 (2)
Sn—Cl1	2.5599 (4)	C6—C7	1.392 (2)
S1—O1	1.5417 (11)	C6—H6	0.9500
S1—C9	1.7796 (17)	C7—H7	0.9500
S1—C8	1.7815 (17)	C8—H8A	0.9800
F1—C5	1.360 (2)	C8—H8B	0.9800
C1—C2	1.494 (2)	C8—H8C	0.9800
C1—H1A	0.9900	C9—H9A	0.9800
C1—H1B	0.9900	C9—H9B	0.9800
C2—C7	1.400 (2)	C9—H9C	0.9800
C2—C3	1.401 (2)		
			
C1—Sn—C1^i^	180.0	C4—C3—C2	121.27 (15)
C1—Sn—O1	95.99 (5)	C4—C3—H3	119.4
C1—Sn—Cl1	90.05 (5)	C2—C3—H3	119.4
C1—Sn—Cl1^i^	89.95 (5)	C5—C4—C3	118.50 (15)
C1—Sn—O1^i^	84.01 (5)	C5—C4—H4	120.8
C1—Sn—Cl1^i^	89.95 (5)	C3—C4—H4	120.8
O1—Sn—Cl1	90.44 (3)	F1—C5—C4	118.88 (15)
O1—Sn—Cl1^i^	89.56 (3)	F1—C5—C6	118.46 (15)
C1^i^—Sn—O1	84.01 (5)	C4—C5—C6	122.66 (16)
C1^i^—Sn—O1^i^	95.99 (5)	C5—C6—C7	118.07 (15)
O1—Sn—O1^i^	180.0	C5—C6—H6	121.0
C1^i^—Sn—Cl1^i^	90.05 (5)	C7—C6—H6	121.0
O1^i^—Sn—Cl1^i^	90.44 (3)	C6—C7—C2	121.47 (15)
O1^i^—Sn—Cl1	89.56 (3)	C6—C7—H7	119.3
Cl1^i^—Sn—Cl1	180.000 (18)	C2—C7—H7	119.3
O1—S1—C9	104.51 (8)	S1—C8—H8A	109.5
O1—S1—C8	102.41 (7)	S1—C8—H8B	109.5
C9—S1—C8	98.73 (8)	H8A—C8—H8B	109.5
S1—O1—Sn	127.03 (6)	S1—C8—H8C	109.5
C2—C1—Sn	115.97 (11)	H8A—C8—H8C	109.5
C2—C1—H1A	108.3	H8B—C8—H8C	109.5
Sn—C1—H1A	108.3	S1—C9—H9A	109.5
C2—C1—H1B	108.3	S1—C9—H9B	109.5
Sn—C1—H1B	108.3	H9A—C9—H9B	109.5
H1A—C1—H1B	107.4	S1—C9—H9C	109.5
C7—C2—C3	118.03 (15)	H9A—C9—H9C	109.5
C7—C2—C1	121.12 (14)	H9B—C9—H9C	109.5
C3—C2—C1	120.85 (15)		
			
C9—S1—O1—Sn	92.64 (10)	C3—C4—C5—F1	179.52 (15)
C8—S1—O1—Sn	−164.80 (9)	C3—C4—C5—C6	0.4 (3)
Sn—C1—C2—C7	81.29 (17)	F1—C5—C6—C7	−179.60 (15)
Sn—C1—C2—C3	−98.42 (16)	C4—C5—C6—C7	−0.5 (3)
C7—C2—C3—C4	−0.3 (2)	C5—C6—C7—C2	0.1 (3)
C1—C2—C3—C4	179.42 (15)	C3—C2—C7—C6	0.2 (2)
C2—C3—C4—C5	0.0 (2)	C1—C2—C7—C6	−179.50 (15)

Symmetry code: (i) −*x*+1, −*y*+1, −*z*+1.

### Hydrogen-bond geometry (Å, º)

*Cg*1 is the centroid of the C2–C7 ring.

**Table:**

*D*—H···*A*	*D*—H	H···*A*	*D*···*A*	*D*—H···*A*
C3—H3···F1^ii^	0.95	2.49	3.333 (2)	147
C6—H6···Cl1^iii^	0.95	2.77	3.6129 (18)	148
C8—H8*B*···Cl1^iv^	0.98	2.76	3.6379 (17)	150
C9—H9*C*···*Cg*1^v^	0.98	2.67	3.3887 (19)	130

Symmetry codes: (ii) −*x*, *y*+1/2, −*z*+1/2; (iii) *x*, −*y*−1/2, *z*−1/2; (iv) −*x*+1, *y*−1/2, −*z*+1/2; (v) *x*+1, *y*, *z*.
